# Entomophagy in Latin America and its potential for sustainability and food security

**DOI:** 10.3389/fnut.2026.1777578

**Published:** 2026-03-18

**Authors:** Reyna Ivonne Torres-Acosta, Gilberto Ruiz-De-La-Cruz, Sugey Ramona Sinagawa-García, Antonia Hernández-Trejo, Carlos Hurtado-Noriega, Gabriel Gorrín-Armas, Maribel Mendoza-Alatorre, Josselyn Paulina Pico-Poma, María Cruz Juárez-Aragón, Edward Alexander Espinoza-Sánchez, Héctor Jaime Gasca-Álvarez, Brenda Julián-Chávez, Diego Abelardo Sarabia-Guevara, Jorge Ariel Torres-Castillo, Eleazar Benítez-Martínez, Luis Daniel García-García

**Affiliations:** 1 Universidad Autónoma de Tamaulipas, Unidad Académica Multidisciplinaria Mante Centro, Ciudad Mante, Tamaulipas, Mexico; 2 Universidad Autónoma de Tamaulipas, Unidad Académica Multidisciplinaria Río Bravo, Ciudad Río Bravo, Tamaulipas, Mexico; 3 Universidad Autónoma de Nuevo León, Facultad de Agronomía, General Escobedo, Nuevo León, Mexico; 4 Universidad Científica del Sur, Lima, Peru; 5 Centro de Estudios Parasitológicos y de Vectores (CEPAVE) (UNLP-CONICET), La Plata, Buenos Aires, Argentina; 6 Laboratorio de Biotecnología I, Facultad de Ciencias Químicas, Universidad Autónoma de Chihuahua, Chihuahua, Mexico; 7 Departamento de Procesos Industriales y Alimentarios, Universidad Estatal Amazónica, Puyo, Ecuador; 8 Universidad Autónoma de Tamaulipas Instituto de Ecología Aplicada, Ciudad Victoria, Tamaulipas, Mexico; 9 Grupo de Ecología de Organismos GEO, Programa de Biología, Universidad Pedagógica y Tecnológica de Colombia, Tunja, Boyacá, Colombia; 10 Programa de Investigación Corporación Sentido Natural, Bogotá, Colombia; 11 Tecnológico Nacional de Mexico, Instituto Tecnológico de Ciudad Victoria, Ciudad Victoria, Tamaulipas, Mexico

**Keywords:** anthropoentomophagy, bioculture, biofunctional, nutrition, sustainability

## Abstract

Entomophagy is often considered as an alternative and complementary practice in human food security. Recently, the exploration of edible insects has taken an increased focus on traditional practices, thus highlighting the importance of inherent folklore in the search of protein-rich food alternatives and their biofunctional properties. The present work summarizes edible insect species in Latin America, some of which are well known and commercialized species. However, most of these Latin-American species are directly extracted from the ecosystem, thereby entailing a complex interaction between sociocultural practices, biodiversity, and food requirements. Latin America possesses an extensive culture concerning the use and consumption of insect species. Therefore, previous experience regarding the mass production and commercialization of said species, along with their utilization as sustenance, constitute a sound reference frame in the exploration of wild or less consumed species, thence improving food security resources.

## 1 Introduction

Entomophagy, or the consumption of insects as a source of sustenance for humans, has gained relevance thanks to its food security potential and environmental sustainability, both of which constitute serious global challenges (1, 2). Insects are a rich source of nutrients, thus making them a viable alternative to other traditional sources of animal protein (3), which is considered an option to current livestock systems compromised by excessive use of natural resources (4–6). However, an insect-based diet, although considered a protein source alternative, is heavily influenced by cultural and societal norms and behavioral trends (7–9). Regardless, entomophagy has become diversified despite being initially considered a traditional practice whose strong attachment to territorial and biocultural aspects relies on availability and ancestral knowledge. Notably, current insect-based formulations are rooted in real nutritional advantages and have been highlighted as suitable supplements for athletes (10, 11). It is, indeed, this relative impact on food production that constitutes one of the key positive aspects of an insect-based diet because, as the human population increases, protein demand becomes more intensive (12), which could also imply a greater environmental impact. In this regard, the production of edible insects (EI) has become a viable option, since production can be supported through agro-industrial residues (13–15) by means of forestry management.

Available literature indicates marked differences in feed-to-meat conversion efficiency among animal production systems, with approximately 10 kg of feed required to produce 1 kg of beef, 5 kg for pork, and 2.5 kg for chicken. In contrast, the production of 1 kg of house cricket biomass has been reported to require as little as 1.7 kg of feed, suggesting a lower environmental impact, although current production costs remain comparatively higher (16–19). Although there are serious arguments against the utilization of EI, these mostly arise from rearing conditions, used substrates, and the species in question; therefore, the exploration of adaptable species to markets and regional production issues should be underscored (18). Evidently, at a global level, few species have been reared on a massive scale due to technical feasibility, legal issues, market trends, and cultural barriers (20, 21). Nevertheless, there are several species with potential to offer a repertoire of nutritional value, flavor, aroma, and texture in regions where insect consumption is rather extensive (21). The objective of this mini-review is to contribute to the analysis of the current state of entomophagy in Latin America by integrating biological, cultural, and sustainability perspectives. It focuses on representative native edible insect species, highlighting their modes of use and production, and examines the main challenges related to sustainability, farming potential, and regulatory frameworks, within the biocultural context of insect consumption and its potential for food security and sustainable food systems.

## 2 Methods

Information on native EI in Latin America was compiled through searches in Google Scholar and NCBI using keywords such as “entomophagy,” “edible insects,” and the scientific names of selected species. The literature was selected based on relevance to discussions on sustainability, food systems, and cultural dimensions, including both well-documented taxa and less explored species. As a narrative review, this work does not aim to provide an exhaustive or systematic assessment of the literature, and its synthesis is inherently qualitative and interpretative.

## 3 Representative edible insects exploited in Latin American

**Figure 1:**
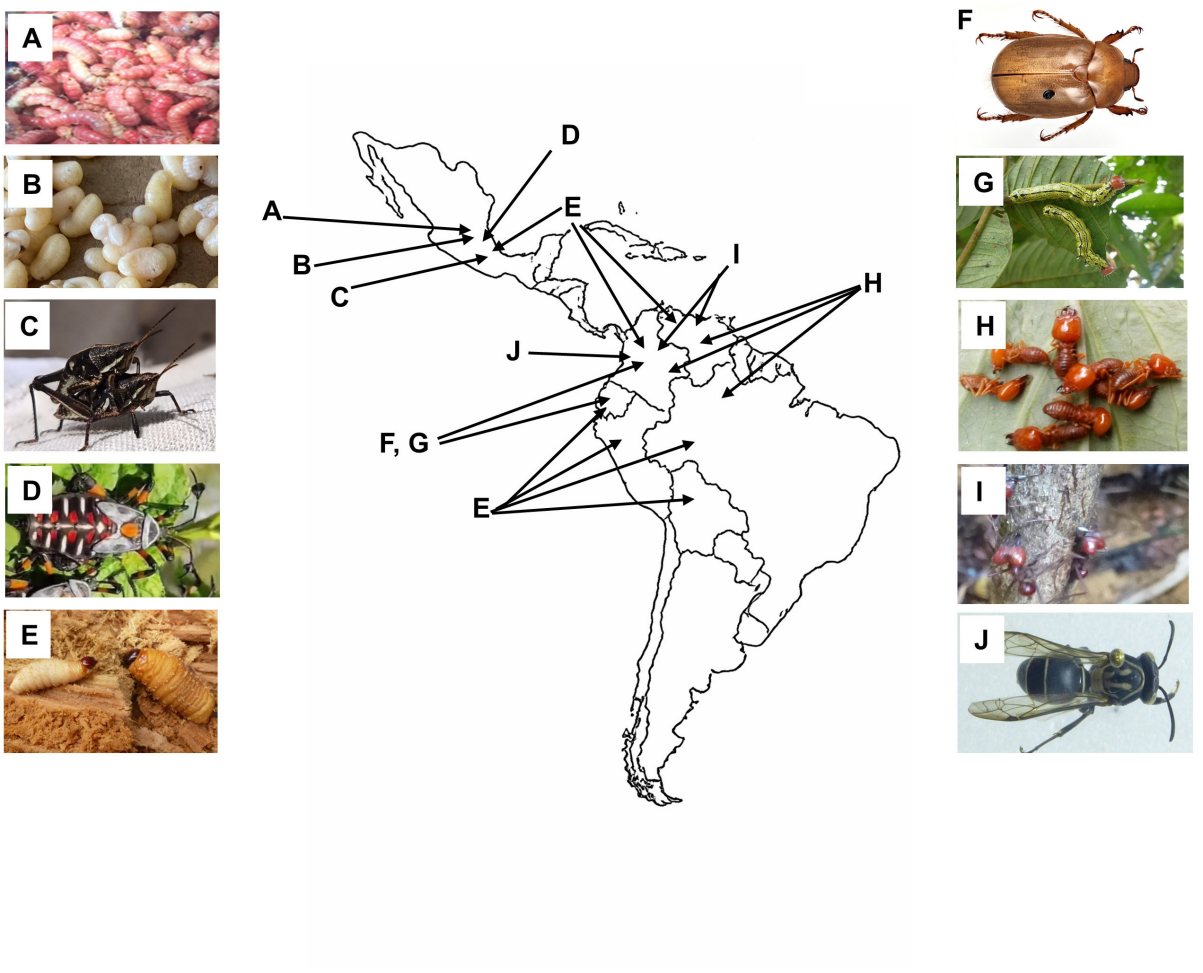
Representative images of edible insects from this study and regions in Latin America where they are consumed. **(A)** Larvae of *Comadia redtenbacheri* (Red maguey worm), adapated from nuevo226 (2758910), iNaturalist Mexico, licensed under CC BY-NC 4.0. Image cropped and contrast adjusted]. **(B)** Escamoles, larvae and pupae of *Liometopum apiculatum* (image by María Cruz Juárez-Aragón). **(C)** Adults of *Sphenarium purpurascens* (grasshoppers) (image by Luis Daniel García-García). **(D)** Nymphs of *Thasus gigas* (Xamues) (image by Gilberto Ruiz-De La Cruz). **(E)** Larvae of *Rhynchophorus palmarum* (Black palm weevil or mojojoy) (image by Héctor Jaime Gasca-Álvarez). **(F)** Adult of *Platycoelia lutescens* (Catzo blanco or white catzo beetle) (Printed with permission from © M. J. Paulsen, Nebraska University). **(G)** Larvae of *Lusura chera* (Tapurú) (image by Héctor Jaime Gasca-Álvarez). **(H)** *Syntermes spinosus* (Manivara) (image by William González). **(I)** Adults of *Atta laevigata* (Leaf-cutter ant) (image by Héctor Jaime Gasca-Álvarez). **(J)** Adult of *Polybia liliacea* (Wasp) (image by William González).

Here, we focused on some native EI used in the Latin American region (Figure 1), candidates for consideration in sustainable management, seasonal use strategies and conservation, leading to the support of food sovereignty and the preservation of traditional knowledge. Some of these species have been explored for bromatological contents although its use is supported by traditional practices (Table 1).

**Table 1:** Representative edible insect species consumed in Latin America and their nutritional and ethnobiological characteristics.

**Species**	**Regional name**	**Order**	**Nutritional contents**	**Season availability**	**Region of consumption**	**Form of preparation**	**References**
*Comadia redtenbacheri*	Red maguey worm	Lepidoptera	Protein: 31.2% Lipids: 58.5% Carbohydrates: 19.02% Ashes: 0.87%	Rainy season (June to September)	Mexico	Snacks, powders, or condiments, fried or grilled	(22, 23)
*Liometopum apiculatum*	Escamole ant	Hymenoptera	Proteins: 42.12–50.63% Lipids: 30.27–34.96% Soluble carbohydrates: 6.80–18.27 Ashes: 6.53–7.85%	February to May	Mexico	Cooked with chili, spices and butter or oil^*^	(24)
*Sphenarium purpurascens*	Chapulín (grasshopper)	Orthoptera	Protein: 50% Lipids: 11% Carbohydrates: 15%−30% Ashes: 1.5%−3.5%	May to December	Mexico	Fried with species, mainly chili, milled and mixed with salt^*^	(25)
*Thasus gigas*	Xamues	Hemiptera	Proteins: 63% Lipids: 26.7% Carbohydrates: not known Ashes: 1.84%	April	Mexico	Roasted and mixed with *salsa* and *tortillas*	(26–29)
*Rhynchophorus palmarum*	Black palm weevil or mojojoy	Coleoptera	Proteins: 25.5% Lipids: 19.7% Carbohydrates: not known Ashes:0.7 to 1%	All the year	Venezuela, Colombia, Ecuador, Brazil, Peru, Paraguay, and Argentina	Raw larvae, cooked in soups, in a stew, fried, or smoked, grilled brochettes and wrapped in leaves and grilled	(30–33)
*Platycoelia lutescens*	Catzo blanco (white catzo beetle)	Coleoptera	Protein: 23.1%−26% Lipid: 9.6%−14.1% Carbohydrate: 23.9%−30% Minerals: 1.58–1.68%.	October to November	Colombia, Ecuador, Perú	Fried in vegetable oil or pork fat and a mixture of onion, tomato, and salt	(34, 35)
*Lusura chera*	Tapurú	Lepidoptera	Proteins: not determined Lipids: not determined Carbohydrates: not determined Ashes: not determined	August to October	Colombia and Ecuador	Cooked, roasted, raw (live)	(29, 36)
*Syntermes spinosus*	Manivara	Isoptera	Proteins: not determined Lipids: not determined Carbohydrates: not determined Ashes: not determined	All the year	Amazonian region of Colombia, Venezuela, Brasil	Cooked, roasted, raw (live)	(29, 37)
*Atta laevigata*	Leaf-cutter ant	Hymenoptera	Proteins: 3.58% Lipids: 4.98% Carbohydrates: 20.24% Ashes: 2.26%	March and April	Amazonian region from Venezuela and Colombia	Cooked, roasted, raw (live)	(29, 38)
*Polybia liliacea*	Wasp	Hymenoptera	Proteins: not determined Lipids: not determined Carbohydrates: not determined Ashes: not determined	All the year	Amazonian region in Colombia	Cooked, roasted, raw (live)	(29)

### 3.1 Red maguey worm—*Comadia redtenbacheri* (Hammerschmidt)

The red maguey worm (Cossidae), also called chinicuil, is associated with agave plants. Typically, it has a seasonal demand as part of central Mexico's cuisine, and it is customarily added into mezcal bottles as an aesthetic factor for consumers (39). With such high demand, initiatives for mass rearing have recently been established (40), one of which suggests the use of potted agave plants, noting that frequent irrigation promotes larval development with a high degree of success (41); however, more research in needed to increase the production of larvae.

### 3.2 Escamole ant—*Liometopum apiculatum* (Mayr)

Locally known as escamole, *L. apiculatum* (Formicidae) have adapted to arid and semi-arid environments through poorly understood strategies that allow them to survive under constant thermal stress and food scarcity (42). In Mexico, escamoles have been traditionally consumed in northern, northeastern, and central regions (43, 44). *L. apiculatum* larvae host many nitrogen-fixing bacteria, which could be associated with the high protein content of escamoles (42).

### 3.3 Grasshoppers—*Sphenarium purpurascens* (Charpentier)

Grasshoppers comprise a group of phytophagous insects of which the more “primitive” ones (Pyrgomorphidae, Romaleidae) preferentially feed on broadleaf plants, whereas the more “evolved” ones (Gomphocerinae, Acrididae) consume grasses (45). Their high nutritional content has been attributed to their diet, which is mostly obtained from maize, bean, wheat, and alfalfa fields, and complemented with wild grasses. The corn-field grasshopper [*S. purpurascens* (Pyrgomorphidae)] has demonstrated great culinary versatility, and it is frequently consumed roasted and flavored with lime, salt, and chili flakes. It is widely accepted and consumed in the central and south Mexico (46). It must be highlighted that, in Mexico, these insects have pre-Hispanic significance and have been used in medicine, religious rites, and as a source of nourishment.

### 3.4 Xamues—*Thasus gigas* (Kulg)

Common in some areas of Mexico, xamues (Coreidae) feed on shrubby and leguminous plants. They are distinguished by their black color and orange-yellow striped patterns, with occasional white details. They are collected directly from the vegetation and are usually consumed in the nymphal stages (i.e., 2nd to 4th instar), although their consumption is not restricted to this period alone. It must be mentioned, however, that upon reaching older stages these bugs acquire atypical flavor notes, sometimes too astringent to be enjoyable (47). Their harvest can be as high as ~3 tons/year in the state of Hidalgo, Mexico (48). When collected they must be cleaned, “desflemados” (fasted for a period of 12–24 h), and then cooked. This species is consumed across central Mexico, and though the extraction impact has been seriously considered, little has been done to maintain its sustainability.

### 3.5 Black palm weevil or mojojoy—*Rhynchophorus palmarum* (Linnaeus)

*Rhynchophorus palmarum* larvae (Curculionidae) represent one traditional food in the Amazon region with a wide use distribution, constituting a source of protein for indigenous communities. Likewise, the oil produced by these larvae is traditionally used in the treatment of respiratory ailments thanks to its linoleic and linolenic acid content, which possess antimicrobial and anti-inflammatory properties (49). These larvae are consumed across several South American countries (32). The collection method is similar among different ethnic groups in the Amazon basin, which mostly consists in taking the larvae from various species of palms. Several studies remark its importance on the diet of indigenous communities, mostly addressing their traditional use and evaluating different biological factors that could enable sustainable production strategies (36, 50).

### 3.6 Catzo blanco—*Platycoelia lutescens* (Blanchard)

For consumption, adult specimens of *P. lutescens* (Scarabaeidae) are cleared of legs, wings, and elytra, then fried and served with toasted corn (51). In general, the nutritional contents of this species are maintained along batches and geographical locations (35). Having said that, these beetles are usually collected directly from the ecosystem; therefore, its wild population has notably declined due to overextraction, environmental impact, improper pesticide management, and climate change. Hence, the availability of this species as traditional food requires mass rearing strategies that would also alleviate the impact on its wild population (51).

### 3.7 Tapurú—*Lusura chera* (Drury)

Tapurú is the common name of caterpillars used as food by indigenous groups in the eastern Colombian Amazon. These caterpillars are collected from different species of trees (29), in which they are known to cause extensive defoliation (>50%) (52). In Colombia, caterpillars are manually collected and consumed during their abundance period (29). In the Peruvian Amazon, *L. chera* (Notodontidae), locally known as awiwa de huaba, has generally been seen associated with *Inga spp*. (53). The recollection forms part of traditional practices by local indigenous groups remarking the relevance of an equilibrated relation between the humans and ecosystems, and need for conservation ancestral knowledge with potential for ensure food security.

### 3.8 Manivara—*Syntermes spinosus* (Latreille)

The word manivara refers to edible termites (*S. spinosus*; Termitidae), its recollection is a very well conserved practice among the indigenous communities of the eastern Colombian Amazon during most of the year, in the case of Macuna and Cubeo, locals spent several hours to recollect enough termites to ensure their meal (29), which includes individuals of different ages, strengthen social relations. Although it is a recurrent edible resource, its harvest completely depends on natural production in the ecosystem and due to their scarce ecological knowledge, there is no approximation to mass rearing. However, observations on recollection show a respectful relation between communities and the biological cycle and nests of this species (29, 36).

### 3.9 Leaf-cutter ant—*Atta laevigata* (F. Smith)

The leaf-cutter ant, *A. laevigata* (Formicidae), is locally known as “culona or arriera” ant for its large abdomen, specifically the winged females. The latter specimens are collected during their nuptial flight at the beginning of the rainy season and consumed by rural communities. These ants are mainly consumed toasted, although other recipes have been developed over time (54–56). In workshops conducted with community participation, the commercialization of processed leaf-cutter ants has been considered. However, a major constraint is the limited availability of the resource in the environment, remarking the need for adequate management strategies of this species (36).

### 3.10 Wasp—*Polybia liliacea* (Fabricius)

These edible wasps are commonly known as “buboronas” wasp in the eastern Colombian Amazon, from which the inhabitants of the Cubeo communities may give them different names in the local language. *P. liliacea* (Vespidae) specimens are usually collected during the night from wild trees and trees from agricultural plots by burning their nests and subsequent manual collection of larvae and pupae (29, 36). Although this species is recognized as an important local food source, it presents a significant limitation, as its traditional harvesting involves the complete destruction of nests. Under this context, extensive exploitation could compromise resource availability at the local scale. Therefore, its use should not be considered viable on a large scale in the absence of appropriate management strategies, and its exploitation should preferably be restricted to traditional practices carried out by local communities.

## 4 Mass rearing of native edible insects

Some of the native edible insect species considered in this mini-review already show initial approaches toward mass or semi-controlled production, even though their life cycles require highly specific environmental and biological conditions. Nevertheless, most native EI in Latin America are still obtained directly through ecosystem extraction rather than farming systems (57, 58). A representative example is the production of larvae of *R. palmarum* from felled trunks of *Mauritia flexuosa*, which are generated as a by-product of palm fruit harvesting. This production system incorporates traditional knowledge from Kukama-Kukamiria communities and highlights the role of rural families in different stages of production, allowing partial optimization of the process (59). In the case of *C. redtenbacheri*, several mass-production approaches have been proposed, and evidence suggests that larvae produced under controlled conditions exhibit improved characteristics for consumption compared to wild-harvested individuals. However, its production remains technically challenging due to the strict management of host plants and husbandry requirements, indicating the need for further research before large-scale implementation (60). More recently, the mass rearing of *S. purpurascens* has been shown to be feasible under prototype farming conditions using maize and soybean sprouts as feed, with potential scalability and adequate nutritional quality of the resulting biomass (61). Nevertheless, additional studies are required to evaluate its economic, technical, and ecological viability at an industrial scale.

For the remaining species addressed in this review, as well as for most native EI in Latin America, widely documented mass-production systems are still lacking, and initiatives for sustainable management remain limited. To date, the close relationship between local communities, traditional ecological knowledge, and restricted consumer demand has likely allowed the continued extraction of native EI without clearly documented negative impacts on natural populations. However, this situation should not be interpreted as evidence that management or production systems are unnecessary, particularly in scenarios where demand or commercialization may increase.

## 5 Regulatory status of edible insects in Latin America

The legal and regulatory framework for edible insect production and commercialization shows differences between Latin America and other regions of the world. In the European Union, EI are regulated under the Novel Food Regulation (EU) 2015/2283, which establishes strict requirements for species authorization, production systems, food safety, and traceability. Similarly, regulatory approaches have been implemented in Singapore, where EI are subject to pre-market approval and safety assessments by national food authorities. These frameworks, although restrictive, provide legal certainty for industrial-scale production and international trade (62, 63).

In contrast, most Latin American countries lack specific legislation addressing EI as food, resulting in regulatory gaps across the entire value chain, including mass production, commercialization, sanitary control, biosecurity issues, protection of endangered species, and sustainability criteria. In many cases, EI are implicitly treated either as wild fauna or informal food products, which limits their formal market integration and discourages investment in farming systems. Furthermore, the absence of clear regulations raises concerns regarding the extraction of wild populations, where overharvesting could pose conservation risks (58, 64, 65).

This regulatory asymmetry represents one of the major limitations for scaling edible insect production in Latin America, but also represents an opportunity for the development of specific policies that integrate traditional knowledge, biodiversity conservation, food safety, and rural livelihoods.

## 6 Discussion

This mini-review contributes to current discussions on entomophagy by moving beyond a predominantly nutritional or technological focus and situating EI within broader biocultural, ecological, and food system contexts in Latin America. The knowledge and consumption of EI reflect a close relationship between society, nature, and culture, rooted in ancestral practices and a deep understanding of local environments and seasonal biodiversity. In a regional context marked by developing economies and persistent socioeconomic difficulties, the integration of entomophagy is increasingly discussed as a potentially viable strategy for improving diet quality, while also representing a sustainable food alternative and an opportunity to strengthen local industries, particularly in areas with limited access to resources.

From an integrative perspective, the analysis of EI in Latin America reveals four interconnected dimensions that must be addressed in a structured manner: sustainability, farming potential, regulatory frameworks, and cultural relevance, which are directly related to Sustainable Development Goals (SDGs) 2 and 13. Although these dimensions are often discussed independently, their interaction ultimately determines the feasibility of EI as a component of sustainable food systems in the region, with the potential to scale up and impact a broader range of communities.

Regarding sustainability, while traditional harvesting practices are often guided by ecological knowledge and seasonal availability, increased demand or commercialization could place pressure on wild populations (57), particularly for species with complex life cycles or destructive harvesting methods, such as nest-harvested wasps or ants. Therefore, sustainability cannot be assumed solely based on tradition and must be evaluated under future demand scenarios.

In terms of farming and scalability, only a limited number of native species show promising approaches toward semi-controlled or prototype mass production, including *R. palmarum, C. redtenbacheri*, and *S. purpurascens*. However, technical constraints related to specific environmental requirements, and life-cycle control remain major limitations (59–61). These constraints explain why most native species have not transitioned from extraction-based systems to standardized farming models.

Unlike regions such as the European Union or Singapore, where centralized regulations provide legal certainty for edible insect production (62, 63), Latin America lacks harmonized frameworks governing food safety, commercialization, biosecurity, and biodiversity protection. This regulatory gap discourages investment in farming systems and raises concerns regarding the unmanaged extraction of native species.

The cultural and socioeconomic dimension is central in Latin America, where EI are deeply embedded in indigenous and rural traditions, contributing also to identity, social cohesion, and local economies. Any attempt to scale production or formalize markets must therefore integrate traditional ecological knowledge and avoid undermining cultural practices (66). Taken together, these four dimensions highlight that the future of entomophagy depends on coordinated advances in sustainable management, applied research, regulatory development, and cultural preservation.

In this sense, entomophagy impacts some SDGs, especially regarding the fight against hunger and climate change mitigation (67), and it must be added that (68) such expectations will be impossible to meet without an integrated approach for the development of sustainable food systems through low environmental impact strategies. Here is where entomophagy and its promotion play a fundamental role, especially for developing countries, opening options for economic development, improvements in nutrition, perpetuating cultural heritage, and nature conservation.

Across Latin America, entomophagy emerges at the intersection of cultural heritage and biodiversity use within local food systems. Traditional knowledge shapes the selective consumption of EI in close relation to ecosystems, seasonality, and harvesting practices. However, these biocultural practices often operate in the absence of consolidated regulatory or policy frameworks, limiting their visibility and formal integration into broader food systems. Recognizing these interactions is therefore essential to critically assess the potential and constraints of entomophagy in sustainability and food security discussions.

## 7 Conclusions

In conclusion, this mini-review highlights the importance of entomophagy in Latin America as a cultural practice that supplements the dietary needs of indigenous communities, with a strong dependence on biodiversity and ancestral traditions. Only a subset of representative species was addressed in this manuscript; therefore, a wide array of EI remains to be explored, highlighting their potential not only as food sources but also as drivers of sustainable economic development. Future research should focus on lesser-consumed species to promote food security and protein diversification while preserving the biocultural values inherent to Latin America. Finally, we emphasize the need for coordinated policymaking and research strategies that facilitate the integration of entomophagy into modern food systems while recognizing and preserving regional traditional practices and biodiversity.
